# Immunosignaturing Can Detect Products from Molecular Markers in Brain Cancer

**DOI:** 10.1371/journal.pone.0040201

**Published:** 2012-07-16

**Authors:** Alexa K. Hughes, Zbigniew Cichacz, Adrienne Scheck, Stephen W. Coons, Stephen Albert Johnston, Phillip Stafford

**Affiliations:** 1 Biodesign Institute, Center for Innovations in Medicine, Arizona State University, Tempe, Arizona, United States of America; 2 Barrow Neurological Institute, St. Joseph’s Hospital, Phoenix, Arizona, United States of America; University of Pittsburgh, United States of America

## Abstract

Immunosignaturing shows promise as a general approach to diagnosis. It has been shown to detect immunological signs of infection early during the course of disease and to distinguish Alzheimer’s disease from healthy controls. Here we test whether immunosignatures correspond to clinical classifications of disease using samples from people with brain tumors. Blood samples from patients undergoing craniotomies for therapeutically naïve brain tumors with diagnoses of astrocytoma (23 samples), *Glioblastoma multiforme* (22 samples), mixed oligodendroglioma/astrocytoma (16 samples), oligodendroglioma (18 samples), and 34 otherwise healthy controls were tested by immunosignature. Because samples were taken prior to adjuvant therapy, they are unlikely to be perturbed by non-cancer related affects. The immunosignaturing platform distinguished not only brain cancer from controls, but also pathologically important features about the tumor including type, grade, and the presence or absence of O^6^-methyl-guanine-DNA methyltransferase methylation promoter (MGMT), an important biomarker that predicts response to temozolomide in *Glioblastoma multiformae* patients.

## Introduction

The identification of biomarkers for presymptomatic detection of disease and classification of existing disease states could provide a rapid and inexpensive adjunct to standard pathological diagnosis. Researchers continue to search for blood-borne protein biomarker(s) for detection of cancer, but sensitivity remains stubbornly low [1], [2]. Immune surveillance, however, occurs continuously and is quite sensitive to changes antigen profiles [3], [4], [5], [6], [7]. It has been demonstrated that cancer cells elicit a detectable humoral immune response [6], [7]. Antibodies make excellent biomarkers because they are stable in serum, have high specificity and affinity to their cognate antigen, are abundant, and enable retrospective studies. An activated B cell can produce 5000–20,000 antibodies per minute [8], [9] while replicating every ∼70 hr [10] with a lifespan up to 4½ months [11], [12], leading to >10^11^ amplification of a specific signal per week. Antibody-based biomarkers avoid the dilution problem seen with proteomic biomarkers [13], [14] and in are not only highly abundant but can be physically captured at nano- and picomolar affinities. Further, unpurified antibodies are stable, allowing archival samples to be used for testing where RNA or proteins may have degraded [15]. The major impediment to using antibodies as biomarkers has been our inability to deconvolve the dense information contained in antibodies as they exist in a complex milieu [16]. There are >10^9^ separate specificities in the blood of an average adult, more than can be examined individually. Fingerprinting cancer antibodies has worked in the past [17], [18] but this has been an onerous task. We introduced immunosignaturing as a simple and very inexpensive approach to diagnostics. Here we address whether immunosignatures are correlated to biological or clinical classifications.

Cancer cells may elicit the production of antibodies against self-antigens or against neo-antigens [3], [18], [19], [20], [21], [22], [23], [24]. We have no *a priori* way to determine exactly what profile of antigens will be presented by a cancer cell (although analysis of EST libraries may suggest candidates). Thus, creating an epitope or protein microarray capable of detecting cancer-specific antigens would be difficult. Although phage display has been shown to detect antibodies specific to cancer [17], [18], [25], for a number of technical and practical reasons panning is not amenable as a diagnostic tool. We created a single-use microarray composed of 10,000 different random-sequence peptides. We use 20mer peptides that incorporate all possible amino acids, except cysteine which we use as a linker. These 20mers can contain at least 7 typical-sized epitopes. Phage display and epitope microarrays tend to use shorter peptides to prevent cross-talk and maintain specificity; in our case, longer peptides allow us to extend the complexity of our microarray which has comparatively few features. Also, the arrays exhibit extremely high reproducibility so that even small differences in binding between antibody and peptide can be significant. The peptides are printed at very high density, an important consideration when using random peptides to detect antibodies that bind at micromolar or even millimolar affinities [26]. The density of these random-sequence peptides on the surface of the microarray creates an avidity-like affect where the off-rate is slowed by several orders of magnitude, enhancing the weak but reproducible interactions between antibody and the peptide. Patterns become detectable, and are so reproducible that sub-classification of diseases is feasible. Although the array has an effective many-to-many relationship between antibody and peptide, the patterns produced are by no means monotonic; in fact they are quite distinguishable from disease to disease. Thus, the ‘immunosignature’ is defined as the common pattern of binding that is shared by patients with a given disease but not with another disease or with healthy controls [27], [28], [29], [30].

We asked whether antibodies raised against cancer cells might be related to, or serve as a proxy for, clinically useful disease biomarkers. To answer the question, we focused on malignant brain tumors. Although the incidence of brain cancer is relatively low compared to other cancers like breast and lung, *Glioblastoma multiformae* (GBM) is one of the most deadly and aggressive tumors with peaks of incidence in both younger and older populations [31], [32]. The most common malignant brain tumors are the astrocytomas, consisting of grade II, grade III (anaplastic) and grade IV (GBM). Malignant tumors may also arise from the oligodendrocytes and are considered low grade oligodendroglioma (grade II) and anaplastic oligodendroglioma (grade III). There are also mixed oligoastrocytomas (low grade and anaplastic). Menigiomas arise from the arachnoid cells that cover the brain and are typically benign, but can recur. A small percentage of these may progress to higher grades that are more invasive. In fact, patients with any of the tumor types mentioned may present with a high grade tumor initially, or they may progress over time. While the diagnosis of some of these tumors may be relatively straightforward, such is not always the case [33], [34]. Neuropathologists are faced with the challenge of making consistent calls – a non-subjective biomarker panel that can distinguish between these different types and grades of brain tumors would be very useful in eliminating lab-to-lab variance. In this paper we present data that illustrates the performance of our immunosignaturing platform for identifying a variety of brain tumor types and subtypes.

## Results

### Immunosignaturing Can Distinguish GBMs from Other Diseases

**Figure 1:**
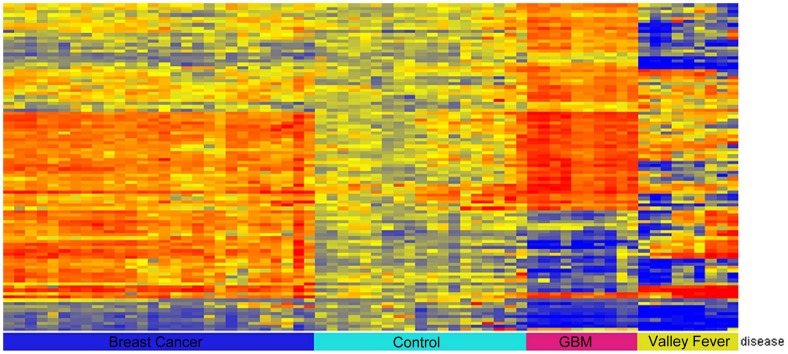
Classification across diseases. The heatmap presented here distinguishes 4 different diseases using 70 peptides identified as the most significant using a 1-way ANOVA across the 27 breast cancer patients, 19 healthy controls, 10 *Gliobastoma multiformae* patients, and 9 Valley Fever patients. Classification accuracy was 100% using both linear discriminant analysis and Support Vector Machines and leave one out cross-validation.

Previously published results from our laboratory [26], [28], [29], [30] have shown signatures for Alzheimer’s disease, infectious disease, monoclonal and polyclonal antibodies. First, we tested the hypothesis that an autoimmune response occurs in patients suffering from brain cancer. In order to do this, we exposed serum from 5 randomly-selected GBM patients from our patient cohort and 3 randomly-selected healthy, age-matched controls to Life Technologies’ ProtoArray®. After controlling for the false discovery rate, no significant reactivity to any protein on the ProtoArray was seen, either for the healthy controls or the cancer patients. Given this, we asked whether cancer was detectable at all on our peptide microarray, and whether different cancer types produced distinct patterns of binding. In fact, cancer and infectious disease both produce a reproducible pattern; Figure 1 shows the 100 most significant peptides selected using 1-way ANOVA with p<1.06×10^−13^ across 28 breast cancer patients, 19 healthy controls, 10 GBM patients, and 9 patients with disseminated *Coccidiodes immitis* (Valley Fever). The classes of disease were simultaneously discernible with 0% leave one out cross-validation error using both linear discriminant analysis and Support Vector Machine when classifying these patients. We conclude that the GBM brain cancer samples have immunosignature patterns distinguishable from other diseases.

### Immunosignature was Reproducible Across Different Sample Sets and Over Time

In 2007, samples from 13 GBM patients and 45 healthy control volunteers were run as a proof-of principal experiment. In 2010, 14 different GBM patients and 13 different healthy controls were run on the immunosignature peptide microarray. In the intervening 3 years, many changes in printing and slide surface chemistry were implemented, so peptide-to-peptide reproducibility improved from an average Coefficient of Variation per array >50% to <15%. Previously we used in-house coated aminosilane slides and a Nanoprint 60 with Telechem SMP2 pins to contact print peptides in a 1-up design. Currently Applied Microarrays (Tempe, AZ) piezo prints peptides onto Schott (Jena, Germany) Nexterion A+ aminosilane-coated slides using piezo deposition to print 10,000 peptides in a 2-up design [26]. However, even with these substantial changes and using new samples, many of the peptides from 2007 had similar classification performance.

**Figure 2:**
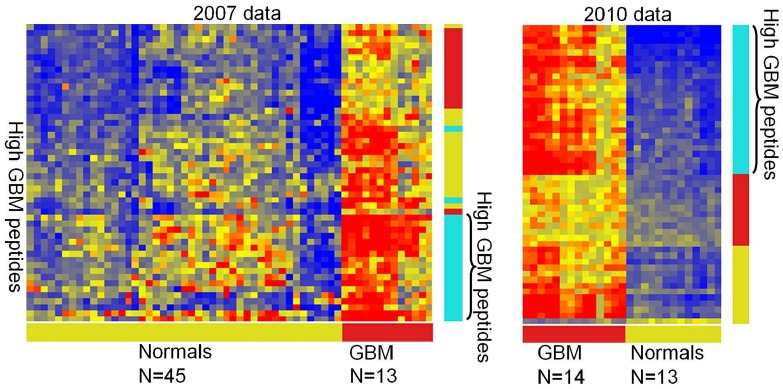
Glioblastoma training and test data. The heatmap on the left shows 50 peptides that differentiated glioblastoma patients from healthy persons obtained from 4 different geographical locations across the US (Fred Hutchison Institute, University of Washington, University of California Irvine, Arizona State University). These peptides were also used to classify different samples consisting of blinded patient and healthy sera obtained from the Barrow Neurological Institute 3 years later. The colored bars on the right indicate clusters that define groups of peptides. Although there are differences between the values obtained in 2007 and 2010, most of the high-binding peptides are very similar.

We found 55 peptides with a t-test p-value <1×10^−6^ between 45 controls and 13 GBM patients (Figure 2, left). We repeated the experiment with 14 GBM and 13 control patients 3 years later with the same peptide sequences (Figure 2 right) and found the new samples had 0% classification error using both LDA and SVM. A k-means clustering was performed on the peptides with k = 3 and found that the peptides that formed the cyan cluster (high-binding in GBM, low in controls) were the same for both the 2007 and 2010 samples.

### Immunosignaturing Can Distinguish Different Brain Tumor Pathologies and Molecular Subtypes

Finally, we asked if pathology of the brain tumor produced a distinguishable immunosignature. We analyzed blood from patients listed in Table 1.

**Table 1:** Patient information and classification performance.

	Astrocytoma grade II	Oligodendroglioma	Mixed astro/oligo	GBM MGMT+^a^	GBM MGMT-b	Control
Total N^c^	23	18	16	16	6	34
Male^d^Female^d^Avg. age^d^	14939±16	81042±13	11546±14	9758±13	3349±14	171742±13
Specificity vs. control^e^	94.1%	100%	100%	vs. MGMT-; 100%	100	
Sensitivity vs. control^f^	91.3%	100%	100%	vs. MGMT-; 100%	100	
Accuracy vs. control^g^	93%	100%	100%	vs. MGMT-; 100%	100	
AUROC^h^	0.927	1	1	1	1	

a = GBMs in which the MGMT promoter is methylated.

b =  GBMs in which the MGMT promoter is not methylated.

c =  Total number of patients with each diagnosis.

d =  number of males and females tested and median age±standard deviation.

e =  classification specificity of that tumor type vs. control.

f =  classification sensitivity of that tumor type vs. control.

g =  classification accuracy of that tumor type vs. control using LDA and LOOCV.

h =  area under the ROC (Receiver Operator Characteristic) curve.

Methylation of the O-6-methylguanine-DNA methyltransferase promoter has been shown to be an important stratifier for GBM. Patients with this molecular subtype have improved survival, particularly when therapy includes temozolomide [38], [39], [40]. Current thinking is that the improved survival is not due solely to this gene, but that methylation of this gene’s promoter may be a marker for a more pleiotropic methylation phenotype.

**Figure 3:**
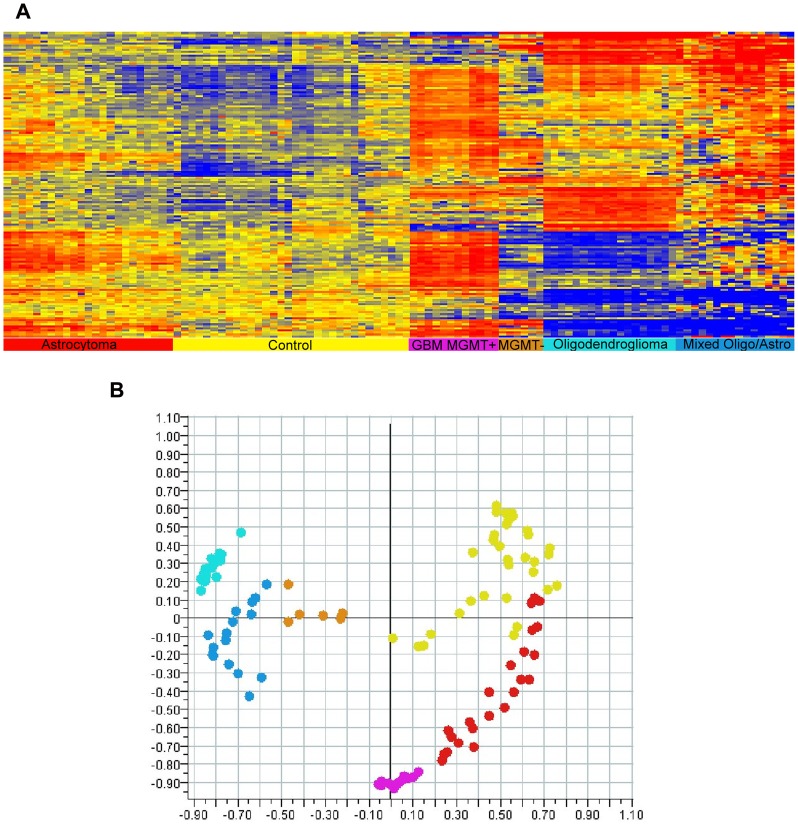
Classification of multiple cancer types and molecular markers. Top: six different classes of brain tumor patients were tested for their immunosignature. We examined *Glioblastoma multiformae* (MGMT- is brown, MGMT+ is purple), astrocytoma grade II (red), oligodendroglioma (cyan) and mixed oligo/astro (blue) against otherwise healthy controls (yellow). We used a 1-way ANOVA to select the 100 most significant peptides, p<10^−18^. High (red) and low (blue) signals correspond to patient antibodies detected with a fluorescently labeled anti-human secondary. Data was grouped using hierarchical clustering on both peptides (Y-axis) and patients (X-axis). Bottom right: principal components display of the separation between samples. X and Y axes represent the first two principal components making up 64% of the total variance across the samples. Patient information is found in Table 1.

The patient samples were run in duplicate on our two-up micoarrays. A technical replicate was run on the top array of one slide and the bottom of another to ensure no top/bottom bias. The immunosignaturing arrays provided a minimum detection limit of 1.25-fold at the 95^th^ percentile across 2 technical replicates on average. Arrays demonstrated a Pearson’s correlation coefficient >0.97 across technical replicates from slides within the same print batch, and >0.91 across print batches. Analysis used the median non-background subtracted signals taken directly from the gpr file (Genepix report). Background subtraction is not used due to the even and very low background. Peptide classification power is calculated using standard power analysis in R [41] (‘power.t.test’ command). For test-training purposes, we split samples 50∶50 randomly, 1000 times and computed the resulting accuracy. For feature selection we use either t-test or ANOVA with appropriate multiple testing correction. 100 peptides whose p values ranged from 10^−27^ to 10^−18^ were used in the heatmap seen in Figure 3.

As evident in the Figure 3a, each of the clinical classifications of brain cancer produced a distinguishable pattern, including the MGMT+ and MGMT- samples. A principal components plot (Figure 3b) shows the relative differences between individual samples, and linear discriminant analysis with leave-one-out cross-validation (Table 1) provides the classification performance and error. Only astrocytoma vs. controls produced mis-classification on the samples tested. We performed test-training using LDA and leave one out cross-validation by evenly splitting the cancer and control samples into test-training sets. We did this 1000 times and obtained 0% error for all pairwise comparisons except astrocytoma vs. control where we obtained an average error rate of 4.7%. We reported an error of 7% in Table 1 which corresponds to a single randomly-selected test-training iteration. We had 0% classification error between GBM MGMT+ vs. controls and MGMT- vs. controls; we also had a 0% classification error between MGMT+ and MGMT- GBM patients. We conclude that the immunosignatures of the clinical classifications are distinct.

## Discussion

We first demonstrated that the immunosignature of GBM was distinct from that of breast cancer and valley fever infection. We also showed that the GBM signature was relatively stable, even over several iterations of the microarray platform. Finally we examined a large number of patients with the most common brain cancer pathologies and found striking immunosignature differences between them. Surprisingly, even GBM patients with differences in O^6^-methyl-guanine-DNA methyltransferase methylation status (MGMT) showed a measurable and common immune signature, enough to classify MGMT status with 0% cross validation error.

An initial concern for the usefulness of immunosignaturing was that the inflammatory response to any disease would dampen specificity for a disease. As shown in our comparison between GBM, breast cancer and Valley Fever, disease specific signatures are evident. A technical concern is the stability of the microarray platform. As shown in Figure 2, GMB-distinctive peptides were evident even on arrays printed 3 years apart with aging (and presumably degrading) solubilized peptide stocks. However, we improved the platform in the intervening years by instituting non-contact printing techniques (Applied Microarrays, Tempe, AZ), larger print batches (136 slides per batch), and automation of sample processing (HS 4800 Pro Hybridization Station, Tecan, Männedorf, Switzerland).

We show for the first time that a pathological state, defining the tumor’s cellular origin or development, can be reflected in the immunosignature (Figure 3a, 3b and Table 1). To our surprise, a single molecular marker, MGMT promoter methylation status, has such a profound effect on the immune system that the immunosignature is distinct. Of course, MGMT promoter methylation may be quite pleotropic and could mark additional changes in the cell causing multiple targets to be presented to the immune system. We do not discount the possibility that the differences we see in immune signatures are due to those multiple proteins being activated by a single promoter. Given that the MGMT status is relevant to outcome and temazolamide response, immunosignatures could lead to a useful non-invasive diagnostic for brain cancer.

In summary, we have shown that immunosignaturing of brain cancer reflects pathological distinctions. Even though the brain is immunologically privileged, cancers can apparently breach the blood-brain barrier and stimulate a broad humoral response. It is important to note that the peptides used on these microarrays are completely random. They can be used for the analysis of any disease in any species; they are *not* specific to brain cancer and they were *not* preselected in any way. Whether immunosignaturing would have diagnostic or prognostic uses for brain cancer is not answered by this study, but the technology is attractive in its simplicity and low cost and has high sensitivity to changes in circulating antibodies. Brain cancer has imposed enormous hurdles that impede detection, monitoring, and treatment. We feel this technology provides a new method to overcome many of these obstacles.

## Materials and Methods

### Samples

Blood was collected from patients undergoing craniotomy for the resection of primary, therapy-naïve brain tumors under IRB# 10BN171 at Barrow Neurological Institute, Phoenix, AZ. Samples were frozen at −20°C from 1 to 7 years prior to use. Blood was centrifuged 100,000×g for 30 min to pellet cell debris and hemolyzed plasma was transferred to a new tube and frozen at −20°C. Healthy volunteers of approximately the same age and male-female composition as the cancer cohort donated blood that was frozen in a similar manner. We also obtained samples from patients diagnosed with breast cancer and Valley Fever. Table 1 shows the number and type of samples used in this analysis. MGMT promoter methylation analysis of GBM tumor tissue was performed by polymerase chain reaction (PCR) analysis of bisulfite-modified DNA as described [42].

### Protoarray Platform: Presence of Autoantibodies

We selected 5 *Glioblastoma multiformae* patients and 3 healthy age-matched controls from our cohort of serum samples. We followed Life Technologies’ protocol for detection of autoantibodies exactly as written. We used Novagen Biologicals (San Diego, CA) biotinylated anti-human secondary at the suggested concentration and Life Technologies’ AlexaFluor 647-Streptavidin as the detecting tertiary. Slides were scanned in an Agilent ‘C’ scanner, 100% laser power, 80% PMT at 10um resolution. Slides were aligned with the appropriate.gal file, and converted to values using Molecular Devices’ (Santa Clara, CA) GenePix Pro 6.0. All controls supplied with the arrays worked as expected; data was analyzed in Agilent’s (Palo Alto, CA) GeneSpring 7.3.1 using FWER = 5%. No proteins on the arrays met the false discovery criteria for significance between healthy vs. cancer patients.

### Immunosignature platform

The immunosignaturing technology has been described elsewhere [26], [27], [28], [29], [30], [35], [36], [37], [43]. Briefly, a 1∶500 dilution of plasma was added to incubation buffer consisting of 5% BSA, 0.1% Tween 20 in 1X PBS pH 7.2. This mixture was incubated on the immunosignaturing microarrays (obtained from www.peptidemicroarraycore.com) at 37°C for 1 hour with agitation on a Tecan 4800 Pro Hybridization Station (Tecan, Salzburg, Austria), dried under molecular grade nitrogen, and scanned on an Agilent ‘C’ scanner at 70% laser power, 20% PMT at 10um resolution, single color mode. 16-bit microarray images were converted to values using GenePix Pro 6.0 (Molecular Devices, Santa Clara, CA) to produce a gpr file. Data was analyzed with GeneSpring 7.3.1 (Agilent, Santa Clara, CA). The Pearson’s Correlation Coefficient across these samples averaged >0.92. Samples with correlation coefficients across technical replicates <0.85 were re-run.

### Informatic Analysis

Preprocessing of microarray data consisted of median normalization per slide and log_10_ transformation. T-tests were Welsh-corrected and adjusted for multiple test bias by using a FWER (Family-Wise Error Rate) of 5%; ANOVA likewise. Peptides were analyzed by testing the hypothesis that there were differences in intensity across the brain cancer or other disease cohorts and control patients as a result of the disease status. Peptides that met these criteria were the ‘features’ were used for classification. Classification used linear discriminant analysis (LDA) with leave-one-out cross-validation. Support Vector Machines (SVM) with a polynomial dot product order of 1 and 0 diagonal scaling factor was used with the initial features to ensure that classification performance was not due to the classifier. SVM produced classification errors within 3% of LDA. A Receiver Operator Characteristic (ROC) curve was created and the area under the ROC curve (AUROC) was calculated (Table 1).
